# Supplementary material for: Laser nano-filament explosion for enabling open-grating sensing in optical fibre
Source: Nat Commun. 2021 Nov 3;12:6344. doi: 10.1038/s41467-021-26671-4 (PMC8566495; doi:10.1038/s41467-021-26671-4)
Supplement: Supplementary file 1 — Supplementary Information [file 41467_2021_26671_MOESM1_ESM.pdf]

Supplementary Information

Laser Nano-Filament Explosion for Enabling Open-Grating Sensing in Optical Fibre

Keivan Mahmoud Aghdami[1,2,3*], Abdullah Rahnama[1,3*], Erden Ertorer[1], Peter R. Herman[1]

[1] *Department of Electrical and Computer Engineering, University of Toronto, 10 King's College Rd., Toronto ON, M5S 3G4, Canada*

[2] *Department of Physics, Payame Noor University (PNU), P.O. Box: 19395-4697, Tehran, Iran*

[3] *These authors contributed equally:* Keivan Mahmoud Aghdami, Abdullah Rahnama.

* To whom correspondence should be addressed: abdullah.rahnama@mail.utoronto.ca

k_aghdami@pnu.ac.ir

Supplementary Fig. 1: Simulated spectra of hollow-array fibre Bragg gratings under varying hole diameter and sensing liquid

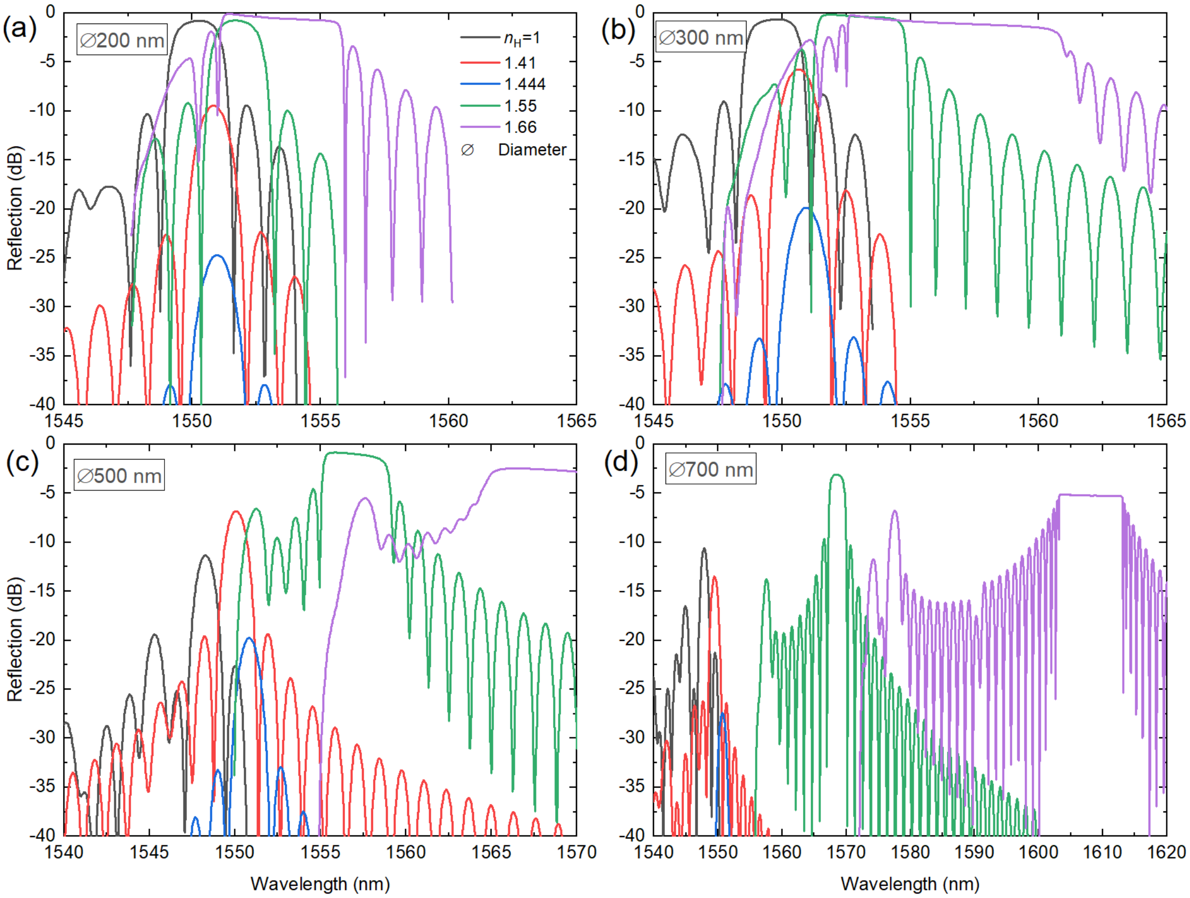

Simulated reflection spectra (EME) generated for second order FBGs (Λ = 1072 nm) having 600 nano-holes spanning over 0.643 mm length. The relatively strong (up to -0.6 dB) and broad (1.2 nm to 11.1 nm) stopbands are noted to shift rapidly with wavelength as shown for the selected examples of air, solvents and oils (n_H = 1.0 to 1.66) applied to fill the nano-holes. The further influence of nano-hole diameter (200 to 700 nm in (a) to (d)) demonstrates the potential of bandgap engineering in generating the strongest stopbands with the smaller range of nano-holes (200 to 300 nm). Alternatively, the largest wavelength shifts arise from the widest hole diameters (700 nm), suggesting a higher RI response. A wavelength shift of +62 nm is noted for at $n_H \cong 1.66$ with nano-hole diameter of 700 nm (d). By tuning the hole diameter, the simulated spectra could be closely matched with the experimentally recorded FBG spectra (Fig. 5f), providing an independent means from SEM in confirming the nano-diameters. Moreover, the close match of spectra confirms the precision of laser-driven filament explosion in providing highly reproducible holes of extra-ordinary aspect ratio on exceptionally close packing densities.

Supplementary Fig. 2: Fibre proof test for mechanical tensile strength with embedded nano-holes

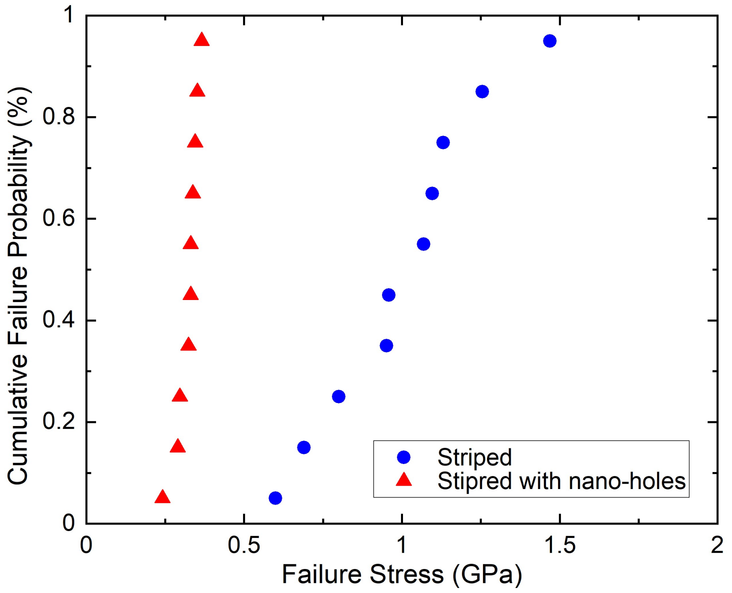

Weibull plot showing cumulative failure probability of tensile strength comparing mechanically stripped SMF-28 fibres embedded with (red triangle) and without (blue circle) a nano-hole array of filament grating. Ten samples have been tested for each fibre type. The FBGs were fabricated with exposures parameters of E_{pulse} = 4.5 µJ, Λ = 1072 nm, and 600 holes, providing strong Bragg resonances of ~-2.5 dB in reflection (i.e., Supplementary Fig. 3) for blind holes penetrating >70 % of the cladding cross-section with diameters of ~200 nm. The mechanical stripping induced damage to cladding as noted by breaking stress values varying over a wide range (0.6 to 1.46) with a median value of 0.95 GPa compared with a pristine fibre having ~5.3 GPa [1]. Fibres with the nanoholes failed withing in a narrow stressing zone of 0.24 to 0.36 GPa, with a median breaking stress of 0.33 GPa lying at approximately one-third of the breaking level for the unmodified fibre. The nanoholes therefore showed relative robustness for a highly structure fibre core and cladding. The data were collected using a commercial fibre proof tester (Vytran, PTR-100). The cumulative failure probability was calculated as a function of stress following the method used in [2].

Supplementary Fig. 3: Effect of filament number on the reflection strength and bandwidth of hollow-array fibre Bragg gratings

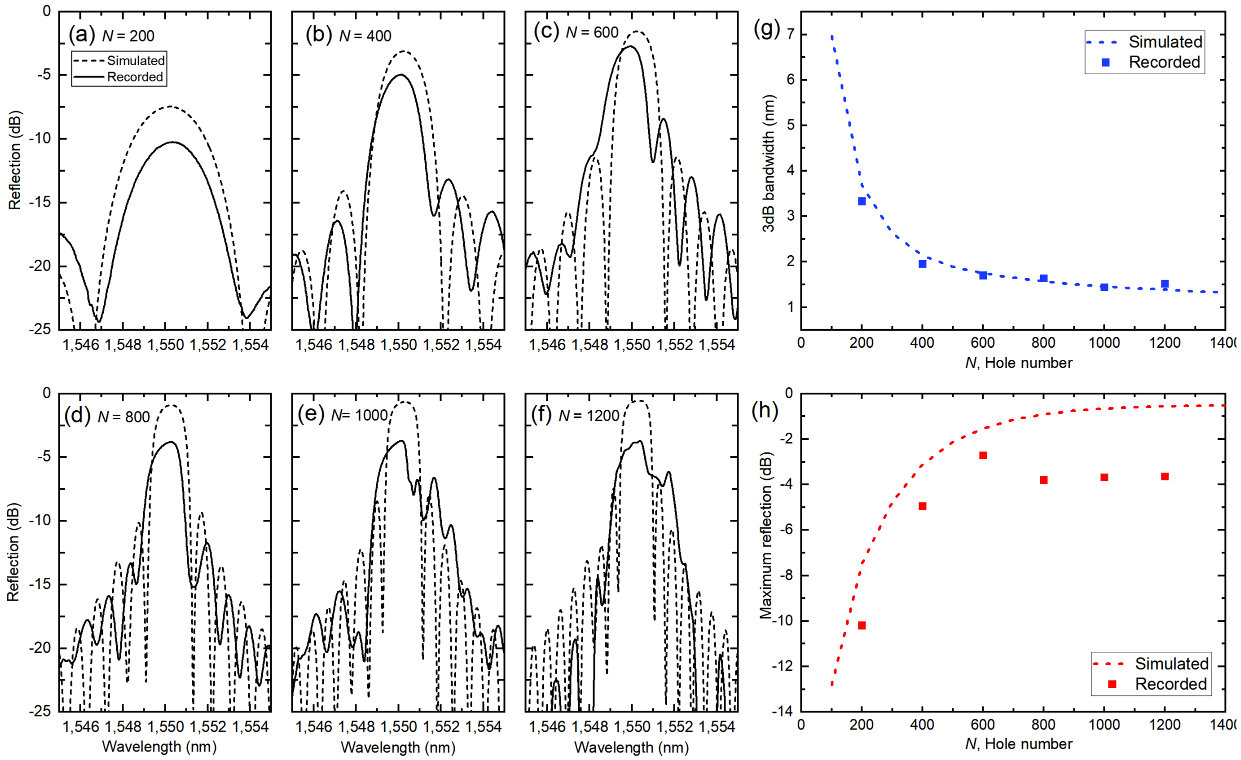

The scaling of FBG reflection strength with the length of filament array is provided by the series of reflection spectra (a-f, solid lines) recorded from second-order gratings (E_{pulse} = 4.5 µJ, Λ = 1072 nm) with air filled holes (n_H = 1.0). A comparison with EME simulated spectra (a-f, dashed line) shows close correspondence up to ~1000 holes, beyond which disorder in the filament sizes or relative positions in laboratory samples (solid lines) appear to accumulate in a broadening, skewing, and mixing of the main lobe peak with the adjacent side lobes. As the number of filaments increases from N = 200 to 1200, the observed bandwidth (g, squares) decreases from 7 nm to 1.5 nm in relative compliance with the EME simulation (g, blue dashed line). The peak reflection (h, squares) falls short by several dB of the simulated values (h, dashed line), increasing from -10 dB at 200 filaments to ~-2.5 dB for 600 filaments. Because gratings with reflection strengths greater than -2.7 dB could not be generated with the present apparatus for air-filled holes, all grating lengths were limited to a maximum of 1.286 mm (i.e., 1200 filaments).

Supplementary Fig. 4: Tuning of Bragg resonance in hollow-arrayed fibre gratings by chemical etching

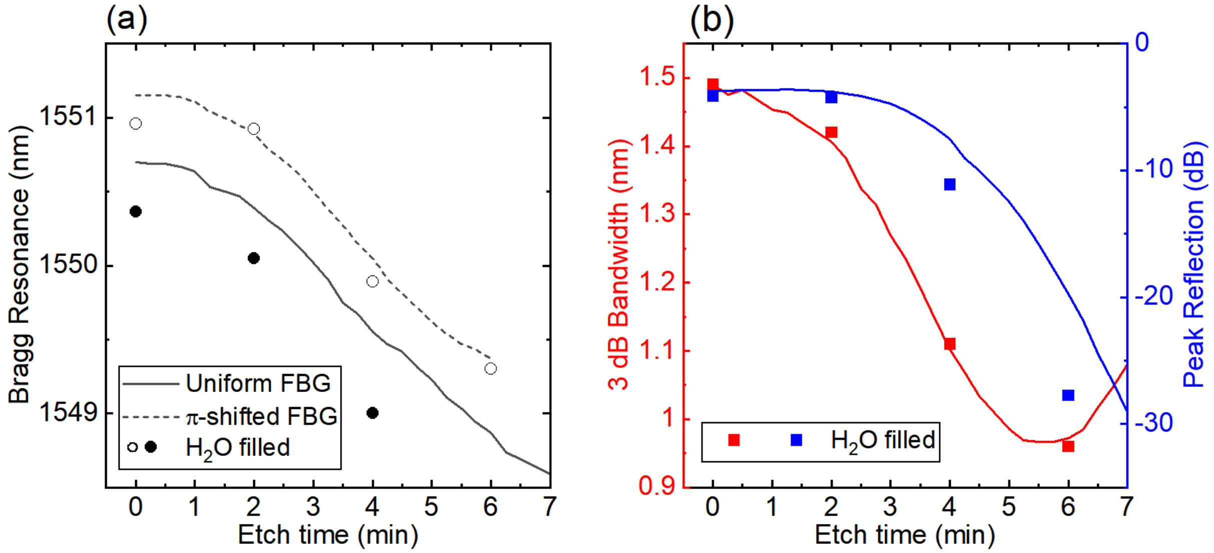

Real-time recording of the influence on the Bragg grating resonance wavelength during chemical etching of second order nano-holed FBGs (E_{pulse}=4.5 μJ, Λ = 1072 nm) with 5% diluted HF acid (a), comparing a uniform 600 filament array (solid line) with a π-shifted array of 1200 filaments (dashed line). Similar spectral shifts are noted for the uniform (solid circle) and π-shifted (open circle) gratings that were removed at different etching times of 0, 2, 4, and 6 min and filled with water. The moderately strong ~2 nm shift to shorter wavelength attests to an increasing diameter of the nano-holes when core waveguide glass has been displaced with a lower RI acid (n_{H} =1.36). For the 600-filament array, the increasing hole diameter resulted in a dramatic fall-off of the peak reflectance from -5 dB to ~-30 dB over a 7 min etching time (b, blue line). In contrast, the Bragg linewidth (3dB) sharpened from 1.5 to 1 nm over a 6 min etching time (b, red line). The peak reflection (b, blue squares) and bandwidth (b, red squares) followed similar trends when the 600 filament FBG was removed after 0, 2, 4, and 6 min of chemical etching and filled with water. The dramatic drop in the peak reflectance to 6 min etching time arises from a hole diameter expanding above an optimal ~$\lambda/4n_{\mathrm{eff}}$ diameter for high reflection beginning in the as-formed FBG (0 min). The rising linewidth and weakening FBG observed beyond 6 min of etching points to the present limit of chemical etching with the onset of nano-hole degradation as holes begin to merge into each other.

Supplementary Fig. 5: Spectral recordings of fibre Bragg stop-bands under varying hole diameter and sensing liquid

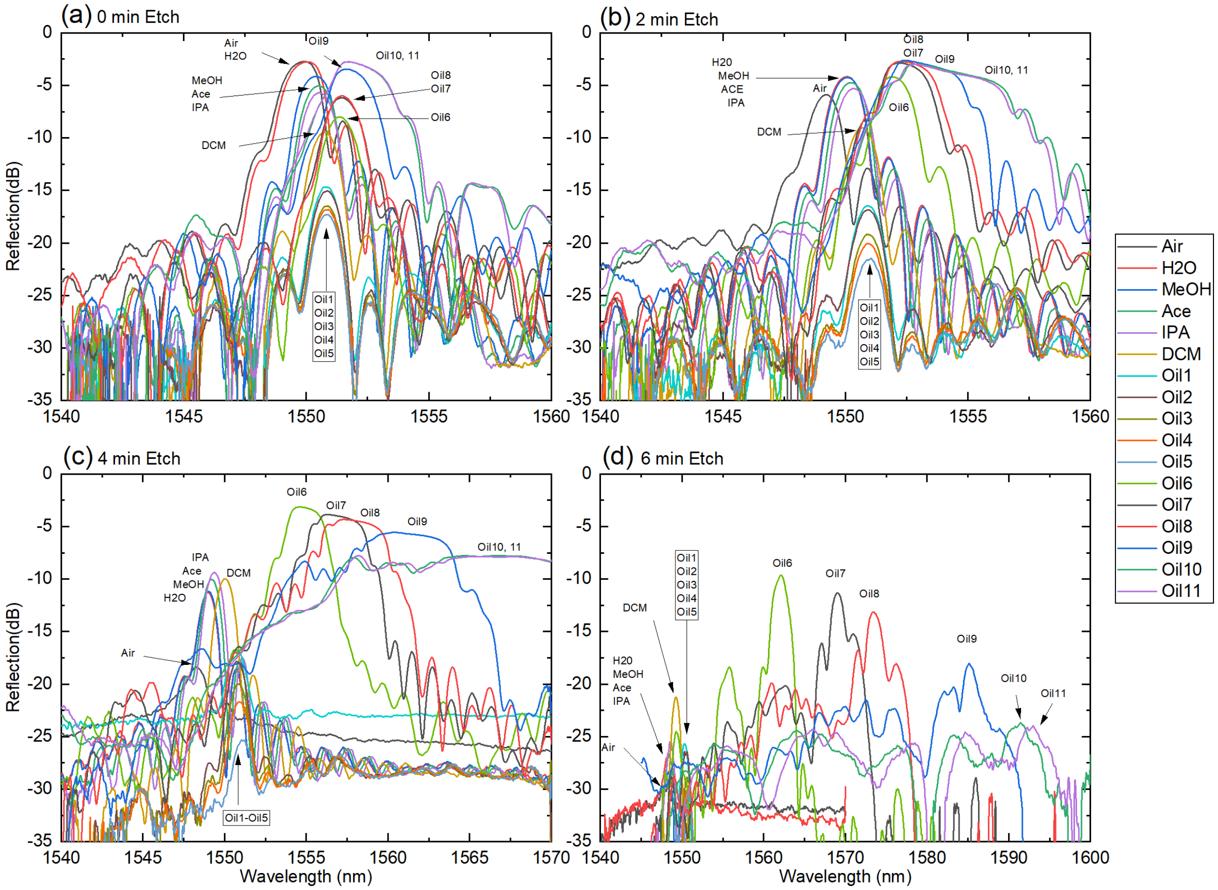

The complete set of the reflection spectra recorded from FBGs having 600 nano-hole filaments (E_{pulse} = 4.5 µJ, Λ = 1072 nm) and their widely varying response to varying RI liquids (colour coded and as labelled in (a)) after undergoing 0 (a), 2 (b), 4 (c) and 6 (d) minutes of chemical etching time. The data complement the representative samples of spectra provided in Fig. 4a and 5a-c. The central wavelength, linewidth and peak reflection of the Bragg resonance for each combination of liquid and chemical etching time were extracted from this data set and plotted in Fig. 5d, e, and f, respectively. The data show that the nano-holes with a smaller diameter such as the unetched case in (a) provide the strongest reflection resonances for a majority of the liquids evaluated, while the increasing larger hole diameters (b to d) provided an increasing Bragg wavelength shift for improving refractive index sensitivity, as shown in Fig. 5f (inset).

Supplementary Fig. 6: Modelling the Bragg stop-bands of arrayed nano-hole fibre gratings

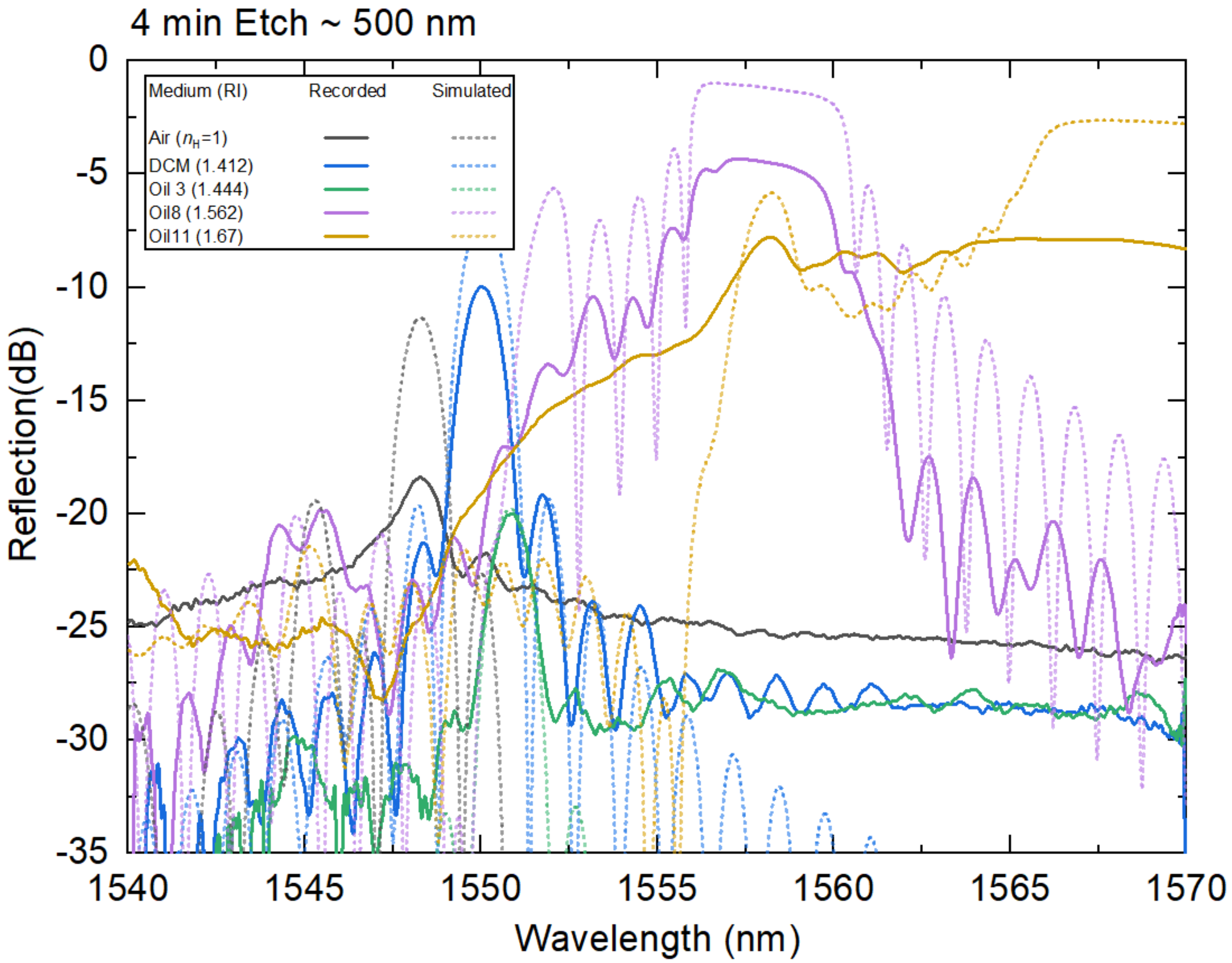

A comparison of experimentally recorded spectra (solid lines) with EME simulated spectra (dashed lines) for uniform FBGs (E_{pulse} = 4.5 μJ, Λ = 1072 nm, 600 holes) that have been chemically etched for 4 min. Only representative values of low to high RI values (n_H = 1.0 to 1.67, see Table 1) have been adopted from Supplementary Fig. 4c. A nano-hole diameter of 500 nm (±10 nm) provided the best alignment of simulated stopband and side-lobe positions to the observed spectra. Relatively symmetric stop bands and side lobes are noted in both of the simulation and experimental data for low to moderate refractive index cases (n_H = 1 to 1.45). In contrast, the positive and high index contrast cases (n > 1.562) have developed an asymmetry that skewed stronger to shorter wavelength. With higher index contrast (Oil11), the short-wavelength band edge forms into a peak separated from a weakening but broadening stopband. An ambiguity in identifying the Bragg shift is thus reached for this case of large hole diameter and high positive contrast in refractive index, leading to a breaking trend of Bragg shifts in Fig. 5 (noted by the hollow triangle data points). With increasingly negative contrast in refractive index (Oil3, DCM, air), the stopbands weaken both in experimental recordings and simulation, that points to a nano-hole diameters of 500 nm approaching the anti-resonant reflection condition approximately at $\lambda/2n_H$ = 775 to 464 nm when n_H = 1.0 to 1.67, respectively.

Supplementary Fig. 7: Experimentally recorded spectra of π-shifted FBGs under varying hole diameter and sensing liquid

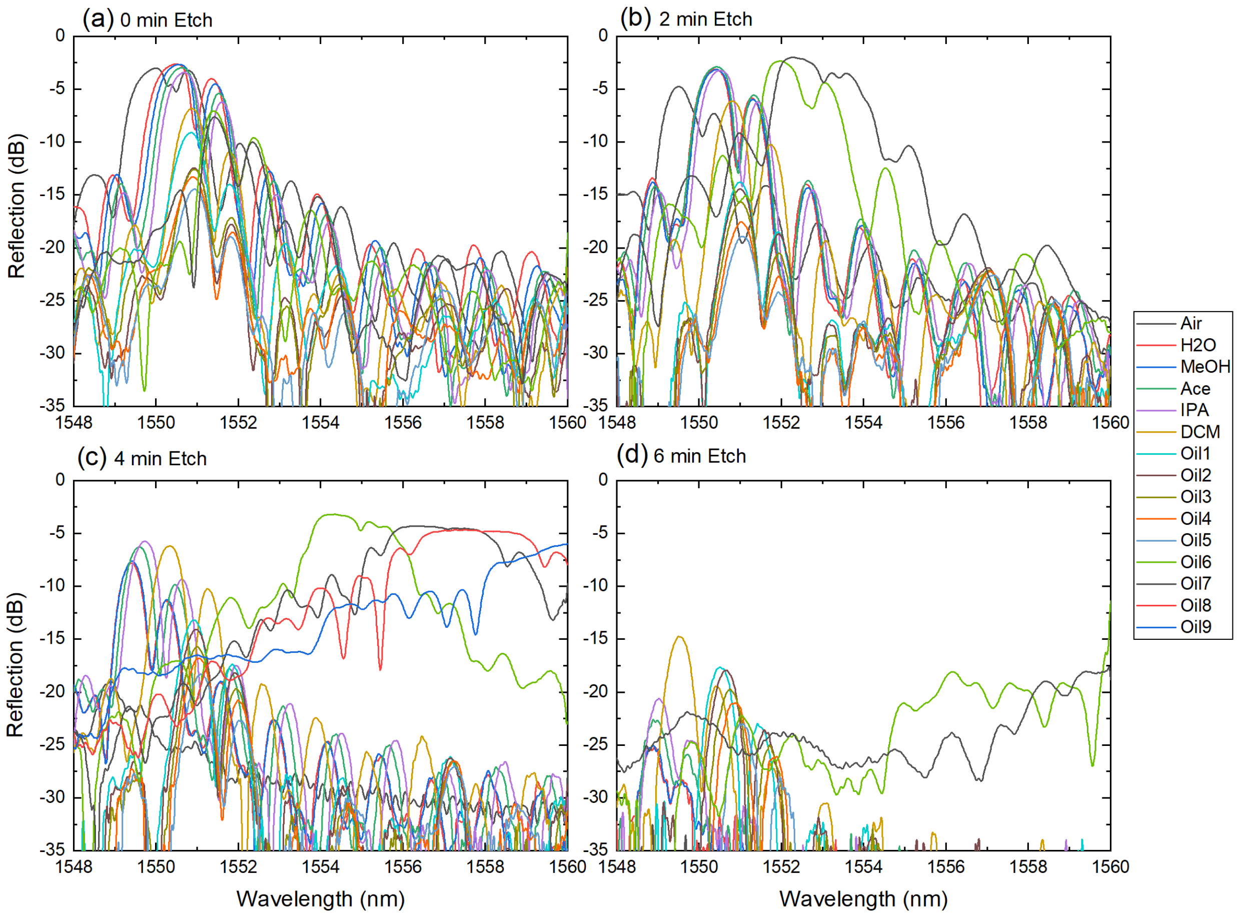

The complete set of the reflection spectra recorded from π-shifted FBGs having 1200 nano-hole filaments (E_{pulse} = 4.5 μJ, Λ = 1072 nm) and their widely varying response to liquids with varying RI values (colour coded and as labelled in (a)) after undergoing 0 (a), 2 (b), 4 (c) and 6 (d) minutes of chemical etching time. Representative samples of spectra were selected from here for presentation in Fig. 4b-e. The Bragg envelop for the present examples of π-shifted FBGs followed identical trends to those as reported for uniform FBGs in Supplementary Fig. 5. Namely, the unetched nano-holes having smallest diameter (a) provided the strongest reflection resonances for a majority of the liquids evaluated, while an increasing hole diameter (b to d) provided an increasing Bragg wavelength shift for improving refractive index sensitivity, but with weakening overall reflection strength.

The π-shift resonance for laser-formed and chemically etched holes appear as narrow (50 to 370 pm) transmission windows that sharpened the sensing resolution of Bragg resonance. However, a birefringent broadening of the π-defect resonance plays out differently in the spectra according the refractive index contrast and hole diameter (See Supplementary Fig. 8 for a full discussion). In the sequence of increasing hole diameter (a-d) for air filled holes (negative refractive index contrast,

$\Delta n =$ -0.45), the π-defect displays a moderately rising birefringence of $\delta\lambda_B$ = 210 to 370 pm for chemical etching times rising from 0 to 6 min. Birefringence broadening of $\delta\lambda_B \cong 20$ pm is also strongly evident in select cases of positive refractive index contrast (i.e., Δn = 0.1 at 2 and 4 min etch for Oil6 and Oil7 in (b) or (c)) Otherwise, the birefringence is unresolved for a majority of the liquid solvents ($1.31 < n_H < 1.67$) and etching time (0 – 6 min) presented here. EME modelling provided further insights into the broadening and birefringence splitting effects as discussed in Supplementary Fig. 8.

Supplementary Fig. 8: Spectral resolution and birefringence response of π-shifted FBGs observed under varying hole diameter and sensing liquid

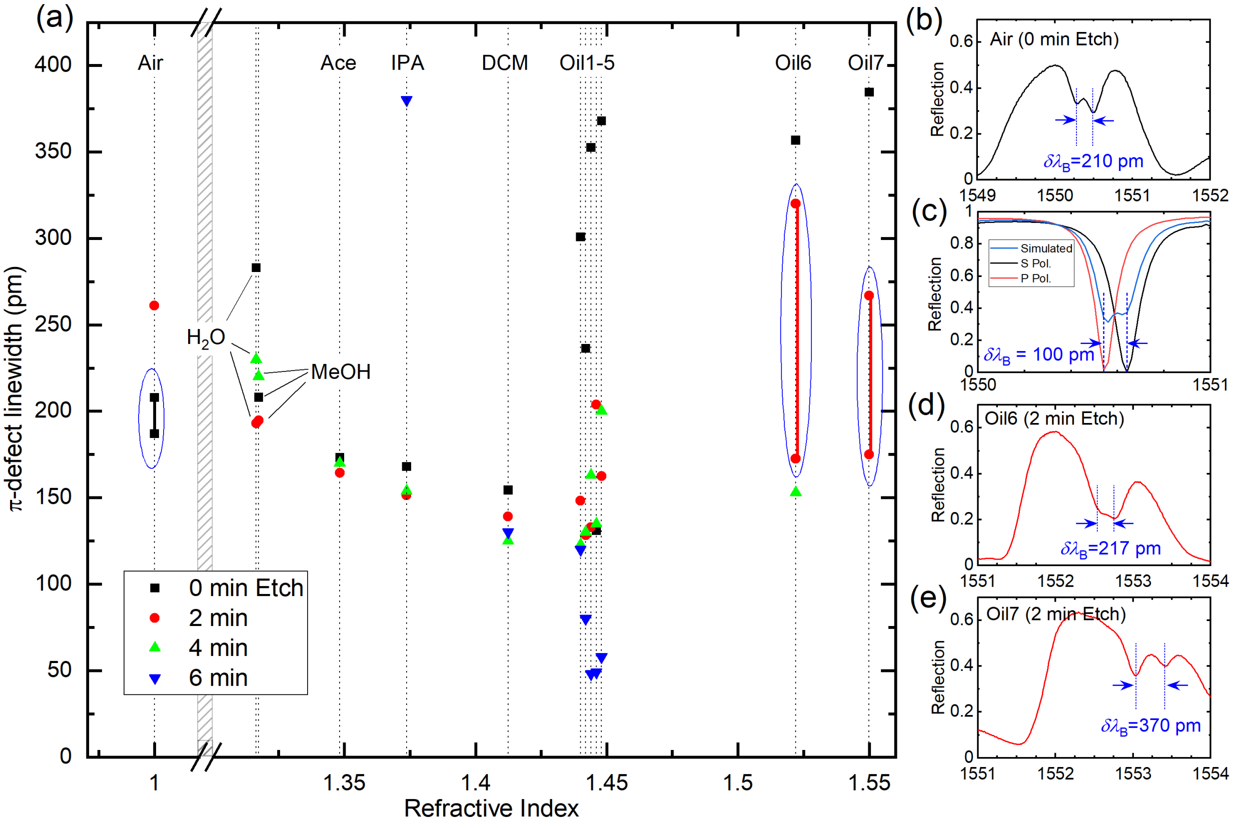

The spectral recordings from π-shifted FBGs (6; 1200 nano-holes, E_{pulse} = 4.5 μJ, Λ = 1072 nm) revealed narrow defect linewidths varying from 50 to 370 pm for unpolarized light as plotted (a) for chemical etching times of 0 (black square), 2 (red circle), 4 (green triangle) and 6 (blue triangle) minutes. Centre wavelengths and 3 dB linewidths were obtained by Gaussian line shaping representations of the π-defect (see Methods). In this way, centre wavelengths and linewidths of the π-defects were followed with ±10 pm and ±5 pm precision, respectively. The sharpest π-defect resonances of 50 to 120 pm (a) were provided by holes filled with solvents having RI values (n_H= 1.440 to 1.448) slightly below that of the core waveguide. In these weaker stopbands, the π-defect narrowed to a minimum 50 pm linewidth as noted for the case of largest hole diameter, inferred to be around 700 nm (Fig. 5f) for the 6 min etching time. The EME modelling predicted an 135 pm linewidth with a birefringent wavelength splitting of 100 pm that closely matched with the observations. Otherwise, higher refractive index contrast generated much broader defect lines of up to 320 pm.

A large component of the π-defect broadening can be attributed to birefringent responses intrinsic to the nano-hole geometry and less so to radial stresses induced by the laser micro-explosion. This birefringence splitting was resolved in only a few of the recorded reflection spectra (Supplementary Fig. 7) which have been reproduced in (b) for an air-filled, non-etched FBG (0

min) with Δn = -0.45 contrast, and in (d) and (e) for a 2 min etched FBG filled with Oil6 (Δn = +0.072) and Oil7 (Δn = +0.100), respectively. Examples of EME simulated spectra (c) for P (red line) and S (black line) polarization show a close alignment in centre wavelength positions with the recorded unpolarized light spectrum (blue line). The simulation provided a wavelength birefringence of $\delta\lambda_B = \lambda_S - \lambda_P = 100$ pm that underestimates the observed $\delta\lambda_B$ = 210 pm as determined by Gaussian line fitting (b). Wavelength birefringence of $\delta\lambda_B$ = 217 and 370 pm as observed for Oil6 and Oil7, respectively, could not be definitively matched with simulated spectra. Since only stress-free nano-holes were simulated in the EME, the radial stresses expected around the nano-hole wall [3] as well as surface tensions effects may play underlying roles.

A narrowing of the π-defect resonances is an objective to further sharpen control of the FBG design that entails improving the nano-hole morphology and alignment positioning in the fibre core.

Supplementary References

[1] Bernier, M., Trépanier, F., Carrier, J. and Vallée, R. High mechanical strength fiber Bragg gratings made with infrared femtosecond pulses and a phase mask. Opt. Lett. **39**, 3646–3649 (2014).

[2] Gu, X., Guan, L., He, Y., Zhang, H. B. and Herman, R. High-strength fiber Bragg gratings for a temperature-sensing array. IEEE Sens. J. **6**, 668-671 (2006).

[3] Bellouard, Y., Champion, A., McMillen, B., Mukherjee, S., Thomson, R.R., Pépin, C., Gillet, P. and Cheng, Y. Stress-state manipulation in fused silica via femtosecond laser irradiation. Optica **3**, 1285-1293 (2016).
