# Supplementary material for: Laser nano-filament explosion for enabling open-grating sensing in optical fibre
Source: Nat Commun. 2021 Nov 3;12:6344. doi: 10.1038/s41467-021-26671-4 (PMC8566495; doi:10.1038/s41467-021-26671-4)
Supplement: Supplementary file 3 — Description of Additional Supplementary Files [file 41467_2021_26671_MOESM3_ESM.pdf]

Description of Additional Supplementary Files

File name: Supplementary Movie 1
Description: Optical microscope video recordings of the optical fibre crosssection (125 µm diameter) have resolved the nano-hole array formed with 200 nm diameter and 1072 nm period in standard optical communication fibre (Corning, SMF-28). The recordings capture the filling of isopropanol into the nano-hole array, with the meniscus confirming that the nano-holes are fully open from cladding-to-cladding without showing hole-to-hole break-through.

File name: Supplementary Movie 2
Description: Complementary recording to Supplementary Movie 1 showing evaporation of isopropanol from nano-holes passing through the silica cladding and guiding core of an optical fibre.
